# Icaritin, an Exogenous Phytomolecule, Enhances Osteogenesis but Not Angiogenesis—An *In Vitro* Efficacy Study

**DOI:** 10.1371/journal.pone.0041264

**Published:** 2012-08-30

**Authors:** Dong Yao, Xin-Hui Xie, Xin-Luan Wang, Chao Wan, Yuk-Wai Lee, Shi-Hui Chen, Duan-Qing Pei, Yi-Xiang Wang, Gang Li, Ling Qin

**Affiliations:** 1 Department of Orthopaedics and Traumatology, The Chinese University of Hong Kong, Hong Kong; 2 The Department of Orthopedics, The First Affiliated Hospital of Soochow University, Soochow, China; 3 Translational Medicine Research and Development Center, Shenzhen Institute of Advanced Technology, The Chinese Academy of Sciences, Shenzhen, China; 4 Stem Cell and Regeneration Program, School of Biomedical Sciences, Faculty of Medicine, The Chinese University of Hong Kong, Hong Kong; 5 Key Laboratory for Regenerative Medicine, Jinan University-The Chinese University of Hong Kong, Ministry of Education of China, Hong Kong; 6 Stem Cell and Cancer Biology Group, Key Laboratory of Regenerative Biology, South China Institute for Stem Cell Biology and Regenerative Medicine, Guangzhou Institutes of Biomedicine and Health, Chinese Academy of Sciences, Guangzhou, China; 7 Department of Imaging and Interventional Radiology, The Chinese University of Hong Kong, Hong Kong; 8 Stem Cells and Regeneration Program, School of Biomedical Sciences, Department of Orthopaedics and Traumatology, The Chinese University of Hong Kong, Hong Kong; Instituto de Engenharia Biomédica, University of Porto, Portugal

## Abstract

We found that Icaritin, an intestinal metabolite of Epimedium-derived flavonoids (EF) enhanced osteoblastic differentiation of mesenchymal stem cells (MSCs) only under osteogenic induction conditions. We also demonstrated its effect on inhibition of adipogenic differentiation of MSCs. Unlike the findings of others on EF compounds, we showed that Icaritin was unable to promote proliferation, migration and tube like structure formation by human umbilical vein endothelial cells (HUVECs) *in vitro*. These results suggested that the exogenous phytomolecule Icaritin possessed the potential for enhancing bone formation via its osteopromotive but not an osteoinductive mechanism. Though some flavonoids were shown to regulate the coupling process of angiogenesis and osteogenesis during bone repair, our results suggested that Icaritin did not have direct effect on enhancing angiogenesis *in vitro*.

## Introduction

A number of bone tissue engineering strategies have been developed in the past to treat bone disorders, such as osteoporosis and steroids-associated osteonecrosis (SAON) [1]. A typical tissue engineering strategy usually involves cells, biomaterial scaffolds, and bioactive molecules [2]. Endogenous growth factors, such as bone morphogenetic proteins (BMPs) and osteogenic protein 1 (OP-1) have been proven osteogenic due to its osteoinductive capability [3]–[5]. Osteoinduction is the process by which osteogenesis is induced, including primitive, undifferentiated and pluripotent cells stimulated to develop into the bone-forming cell lineage [6]. As endogenous growth factors are sometimes biologically unstable and expensive for clinical applications, exogenous biomolecules, such as structurally stable and cost-effective phytoestrogenic molecules from medical herbs have been tested for treatment of musculoskeletal disorders [7]–[10].

Epimedii herba is one of the most widely studied herbs for treatment of bone metabolic and related diseases in China [11]–[13]. Epimedium flavonoid (EF) is composed of seven flavonoid compounds with parent ring and its main active compound extracted from Epimedium is Icariin, which has been reported to enhance bone healing [14] and to prevent osteoporosis and SAON [15]. It has been demonstrated that Icariin stimulates the proliferation of osteoblast-like UMR 106 cells, promotes osteogenic differentiation of rat bone marrow stromal cells (MSCs), improves maturation and mineralization of osteoblasts [16]–[18], and suppresses osteoclastogenesis and inhibits bone resorption activity *in vitro* [19].

Angiogenesis and osteogenesis are tightly coupled during bone development and regeneration [20], [21]. The vasculature supplies nutrients and oxygen to develop and regenerate bone and also delivers critical signals to stroma that stimulate MSCs to differentiate and to enhance bone formation. On the other hand, bone also supplies growth factors and cells to enhance angiogenesis [22]. During bone growth, development, and remodeling, some mediators are identified that couple the angiogenesis and osteogenesis. These include members of the fibroblast growth factor (FGF), transforming growth factor (TGF), bone morphogenetic protein (BMP), insulin-like growth factor (IGF) and platelet derived growth factor (PDGF) families [23]–[25], as well as hypoxia inducible factors (HIFs) and vascular endothelial growth factor (VEGF) [26], [27]. It is also reported that some plant-derived isoflavonoids, such as Genistein and Icariin, could regulate the angiogenesis and osteogenesis [28]–[31].

Our recent study on intestinal metabolism of EF has shown that a small molecule Icaritin is involved in intestinal metabolism of those seven flavonoid compounds with parent ring in EF, which is in accordance to the recent consensus that diversiform isoflavonoids with a parent ring may be intestinally metabolized to a single equal for acting on pharmacological targets [32], [33]; Icaritin may also be involved in enzymatic hydrolysis of prenylated flavonols [34]. Recently, we reported that Icaritin reduced SAON incidence with inhibition of both thrombosis and lipid deposition [35]; others reported that Icaritin could promote osteoblastic while inhibit osteoclastic differentiation [36].

Endogenous growth factors are excellent bioactive molecules for bone tissue engineering with both osteoinductive and osteopromotive effects. However, controversial findings have been reported on the bone formation enhancement by exogenous phytomolecules, such as Icariin. While some studies showed its osteopromotive effect, others reported that such osteopromotive effect was attributed to its osteoinductive potential [37]–[40]. The effects of Icaritin on bone formation were observed but the detailed cellular and molecular mechanisms underlying these effects remained unclear. MSCs are widely used to study their potentials in osteogenic and adipogenic differentiations. MSCs are a multi-potent cell type that gives rise not only to osteoblasts, but also to a range of other cell types, including adipocytes, chondrocytes, and myoblasts [41]. Effects of several different hormones, vitamins, growth factors and cytokines on MSCs proliferation and differentiation have been tested using *in vitro* differentiation assay, studying mainly osteogenic [42], [43], chondrogenic [44], [45] or adipogenic differentiation [46], [47].

In the present study, we explored the underlying cellular and molecular mechanism of Icaritin in regulating osteogenic differentiation of MSCs for understanding its osteoinductive or osteopromotive effect, a scientific foundation for understanding its potentials for enhancement for bone defect repair. We also aimed to examine the effects of Icaritin on angiogenesis *in vitro*, as this would help to clarify whether Icaritin acted directly on angiogenesis during bone repair or indirectly via other factors associated with angiogenic enhancement.

## Materials and Methods

### Ethics

Human fetal bone marrow stem cells were donated from the Stem Cell Bank of the Prince of Wales Hospital of the Chinese University of Hong Kong. Human ethics approval was obtained from the Joint CUHK-NTEC Clinical Research Ethics Committee of the Chinese University of Hong Kong (Reference No. CRE-2011.383). Informed written consent form was approved by the Clinical Research Ethics Committee and signed by donor before sample collection.

### Reagents and Cell Culture

Icaritin was supplied by Institute of Traditional Chinese Medicine & Natural Products in Jinan University; Fibroblast Growth Factor-Basic human (FGF2) was purchased from Sigma (USA). Human bone marrow derived mesenchymal stem cells (BM-MSCs) were kept in Modified Eagle's Medium of Alpha (a-MEM) (Invitrogen) supplemented 10% fetal bovine serum (FBS) (Gibco) and 1% penicillin/streptomycin (Gibco). Human umbilical vein endothelial cells (HUVECs) were purchased from ATCC (American Type Culture Collection, Manassas, VA, USA) and cultured in endothelial cell medium (ECM) (ScienCell TM) (Carlsbad, CA, USA).

### Surface Phenotypes of Human MSCs

1×10∧6/mL MSCs suspension was prepared and washed twice with PBS. 100 µl of cell suspension was resuspended in stain buffer (BD Biosciences, San Diego, CA, USA) and incubated with fluochrome-conjugated primary antibodies against CD34, CD44, CD45, CD73, CD90, CD105, and corresponding isotype control (all antibodies were purchased from BD Biosciences) according to manufacturers' instruction. The stained cells were immediately subjected to flow cytometric analysis using LSRFortessa Flow Cytometry (BD Biosciences, USA).

### Osteogenic and Adipogenic Differentiation of Human MSCs Treated with Icaritin

MSCs were placed into 6-well plate at a density of 10∧4 cells/cm^2^, when over 80% confluence was reached, osteogenic or adipogenic differentiation was induced by the α-MEM containing osteogenic supplements (OS): 10∧-8 M dexamethasone (Dex) (Sigma), 50 µg/ml L-ascorbic acid 2-phosphate (AsAP) (Sigma) and 10 mM glycerol 2-phosphate (Gly) (Sigma) or adipogenic supplements (AS): 10∧-6 M Dexamethasone, 0.5 mM isobutyl-methylxanthine (IBMX) (Sigma), 10 µg/ml insulin (Sigma) and 0.2 mM Indomethacin (Sigma). Icaritin was dissolved in dimethyl sulfoxide (DMSO) and then added during osteogenic or adipogenic differentiation; DMSO served as control. The culture medium was changed every three days and the MSCs were kept under 5% CO_2_ at 37°C in a humidified condition. At the indicated time intervals (i.e. at day 1, 2, 3, 6, 10, 12, 16 and 21), cells were collected for the following experiments: 3-(4,5-dimethylthiazol-2-yl)-2,5-diphenyltetrazolium bromid (MTT) assay; alkaline phosphatase (ALP) staining; ALP activity assay; mineralization assay; Oil Red O staining and Real-time polymerase chain reaction (PCR). Triplicate tests were conducted in each experiment.

### MTT Assay for Proliferation of MSCs and HUVECs

MSCs were placed onto 96-well plates at a density of 5000 cells/cm^2^. After incubation for 24 h, the medium was changed into Icaritin containing medium with different concentrations from 10∧-4 M to 10∧-10 M accordingly, DMSO served as control, the cells then were incubated at 37°C for different intervals at day 1, 2, 3 and 4. For MTT assay, the medium was replaced with 20 µl of MTT (5 mg/mL) and left to incubate for 4 h in dark at 37°C. The supernatant was discarded and 200 µl DMSO was added, then the plate covered with tinfoil and agitated on orbital shaker for 15 min. Absorbance was measured for all samples at 590 nm with a reference filter of 620 nm by VICTOR3 VTM Multilabel Counter (PerkinElmer, USA). For HUVECs proliferation assay, the cells were cultured in 96-well plates with 5000 cells/cm^2^ for 24 h, then the medium was refreshed and Icaritin with different concentrations ranging from 10∧-5 M to 10∧-12 M was added accordingly, FGF2 was dissolved in sterile water and served as positive control, sterile water and DMSO were served as negative controls. After the cells were incubated in 37°C for 1, 2 and 3 days respectively, MTT assay for HUVECs proliferation was performed as described above.

### ALP Staining

After collection of the cells treated with or without OS and Icaritin for 10 days, the cells were washed with PBS twice and fixed with 70% ethanol for 10 min, then equilibrated by 1 ml ALP buffer (0.1 M NaCl, 0.1 M Tris-HCl, 50 mM MgCl_2_•6H_2_O, 0.1% Tween-20, PH 9.5) twice, 0.5 ml ALP substrate solution (5 µl BCIP and 10 µl NBT in l ml ALP buffer) was added to each well of the 6-well plate, incubated at 37°C in dark for 60 min before the reaction stopped by distilled water and then dried before taking photo.

### ALP Activity Assay

Briefly, MSCs were treated with or without OS and Icaritin for 10 days and DMSO served as control. The plate was washed with PBS buffer solution and the cells were lysed by diethanolamine (DEA) lysis buffer (20 mM Tris–HCl (pH 7.5), 150 mM NaCl, and 1% Triton X-100) at 4°C for 15 min, then the cell lysate was centrifuged at 4°C for 5 min and the pellets were discarded. The ALP activity was determined using p-nitrophenylphosphate as the substrate. Sample volumes of 30 µl were added to 170 µl p-nitrophenylphosphate. Absorbance at 405 nm was measured and the protein concentration of cell lysates was measured using the Bradford assay at 595 nm on a microplate spectrophotometer (Bio-Rad, USA). ALP activity was normalized according to the total protein content of cell lysate.

### Alizarin Red S Staining

Calcium deposition was determined by Alizarin Red S staining. Briefly, the cells treated with or without OS and Icaritin for 16 days and DMSO served as control. Cells were then washed with PBS and fixed with 70% ethanol for 30 min, washed with PBS again and stained with 2% Alizarin Red S (pH 4.0) for 5 min. The plate was washed several times by distilled water before taking photos using digital scanner (Epson Perfection V700 Photo, Epson America. Inc., USA).

### Oil Red O Staining

After adipogenic differentiation of MSCs treated with or without AS and Icaritin for 21 days and DMSO served as control, the plate was washed with PBS twice and fixed with 4% formalin for 10 min, and then the formalin was discarded and washed with 60% propylene glycol. The cells were stained with 0.6% Oil Red O in propylene glycol for 2 h at room temperature, then washed with 60% propylene glycol again to discard the redundant Oil Red O and microscopic photos of the stained fat droplets were taken. After photo-taken, isopropyl alcohol was added in the plate and incubated for 1 h at room temperature to elute the Oil Red O. Aliquots of the extracted oil red O were measured with spectrophotometer at 520 nm (Ultrospec 3000, Pharmacia Biotech, USA).

### Migration Assay

The transwell migration assay was performed on HUVECs using 24-Well Cell Migration Assay kit (5 µm, Fluorometric Format, Cell Biolabs, USA). HUVECs cells were starved overnight in Endothelial Cell Medium (ECM) before starting the experiment. The lower chambers were filled with 1 ml of serum free medium while containing 1 µM Icaritin. 200 µL of the HUVECs suspension solution (5×10∧6 cells/ml) was added to the upper chamber. Cells were incubated at 37°C for 12 h to allow cell migration through the membrane and subsequently lysed and detected by CyQuant® GR dye. Fluorescence measurement was performed in a FluoStar Optima fluorescence plate reader with a 485/520 nm filter. DMSO and FGF-2 were also used as negative and positive control, respectively.

### *In vitro* Angiogenesis Assay

The formation of tube-like structures by HUVECs was performed using BD BioCoat™ Angiogenesis System-Endothelial Cell Tube Formation kit (BD, USA) according to the manufacturer's instruction. Briefly, 50 µl of the HUVECs suspension (4×10∧5 cells/ml) with Icaritin was seeded onto each well of the 96-well plate coated with Magrigel. FGF2 and DMSO served as positive and negative control, respectively. Matrigel cultures were incubated at 37°C for 16 h. Tube formation was observed using an inverted phase contrast microscope and images were captured with a video graphic system. The degree of tube formation was quantified by measurement of the length of tubes in six randomly chosen fields from each well using Image-Pro Plus 6.0 (Media Cybernetics, USA).

### RNA Isolation and Real-time PCR

After osteogenic induction of human MSCs by OS with or without Icaritin treatment for 3, 6 and 12 days respectively, RNA was extracted using RNeasy Mini Kit (Qiagen, Valencia, CA, USA), and then reverse transcribed into cDNA using QuantiTect Rev Transcription Kit according to the manufacturer's instruction (Qiagen). Primer sequences were as follows: ALP forward: 5′- ctcccagtctcatctcct-3′, reverse: 5′-aagacctcaactcccctgaa-3′; Collagen type 1 (Col1a) forward: 5′-cactggtgatgctggtcctg-3′, reverse: 5′-cgaggtcacggtcacgaac-3′; Osteopontin (OPN) forward: 5′-gtaccctgatgctacagacg-3′, reverse: 5′-ttcataactgtccttcccac-3′; Bone sialoprotein (BSP) forward: 5′-ggcacctcgaagacaacaac-3′, reverse: 5′-gcccgtgtattcgtactccc-3′; Runt-related transcription factor 2 (Runx2) forward: 5′-acttcctgtgctcggtgct-3′, reverse:5′-gacggttatggtcaaggtgaa-3′; BMP2 forward: 5′-gtatcgcaggcactcaggtc-3′, reverse: 5′-cacttccaccacgaatccat-3′; BMP4 forward: 5′-cgaatgctgatggtcgttt-3′, reverse: 5′-cagggatgctgctgaggtta-3′; Peroxisome proliferator activated receptor gamma (PPAR-γ) forward: 5′- gaaacttcaagagtaccaaagtgcaa-3′, reverse: 5′-aggcttattgtagagtctgagtcttctc-3′; Glyceraldehyde-3-phosphate dehydrogenase (GAPDH) forward: 5′- ggcatggactgtggtcatgag-3′, reverse: 5′-tgcaccaccaactgcttagc-3′. Real-time PCR was performed in 384-well plates using TF pack power SYBR Green PCR Mas (Applied Biosystem, Foster City, CA, USA) and ABI Prism 7700 sequence Detection System (Applied Biosystem). Amplification was carried out for 40 cycles, first at 95°C 10 min for hold, and then each cycle was at 95°C for 15 s, 60°C for 1 min. Quantitative analysis was performed according to the ABI protocol. The threshold cycle (Ct) value was calculated from amplification plots. Relative quantification of gene expression was determined using the delta delta CT (ΔΔCT) method, with each sample being normalized to the expression level of GAPDH.

### Statistical Analysis

All data were expressed as means ± SEM. Statistical analysis was performed using SPSS 17.0 software (Chicago, IL, USA). One-way analysis of variance (ANOVA) followed by Tukey post hoc test (multi-group comparison) was used to assess statistical significance at (*) p<0.05 and (**) p<0.01.

## Results

### Characterization of surface phenotypes of human MSCs

**Figure 1:**
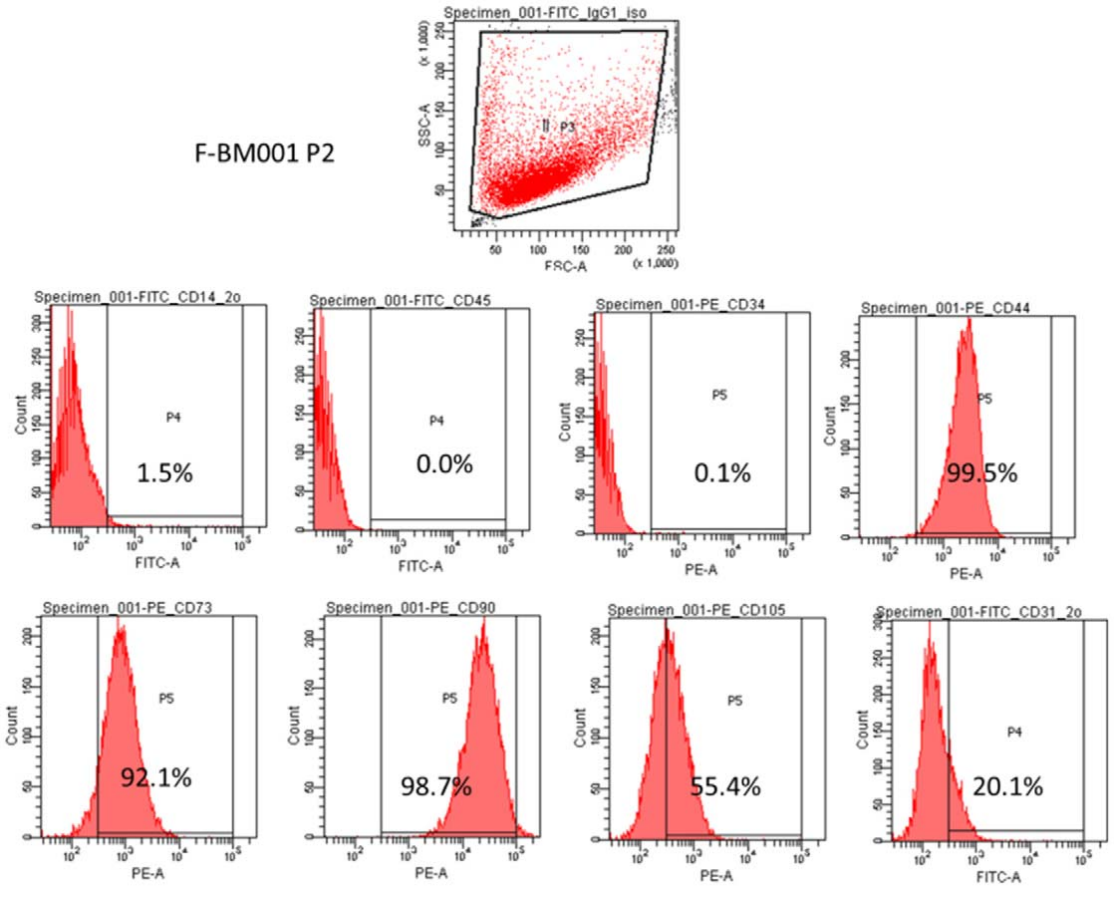
Characterization of human fetal bone marrow derived mesenchymal stem cells. MSCs were collected and wished with PBS, then incubated with fluochrome-conjugated primary antibodies against CD34, CD44, CD45, CD73, CD90, CD105, and corresponding isotype control. The stained cells were immediately subjected to flow cytometric analysis using LSRFortessa Flow Cytometry.

In this study, the mesenchymal stem cell property of the human MSCs was characterized by the presence of surface phenotypes by flow cytometry. Fig. 1 showed that at passage 2, the cells were positive to CD44, CD73, CD90 and CD105 and negative to CD14, CD34 and CD45. The slight presence of CD31 indicated the presence of endothelial cells in this cell population. The flow cytometery data showed that our cultured human MSCs expressed standard surface markers of MSCs and therefore used for experiments described below.

### Icaritin had no effect on human mesenchymal stem cells (MSCs) proliferation

**Figure 2:**
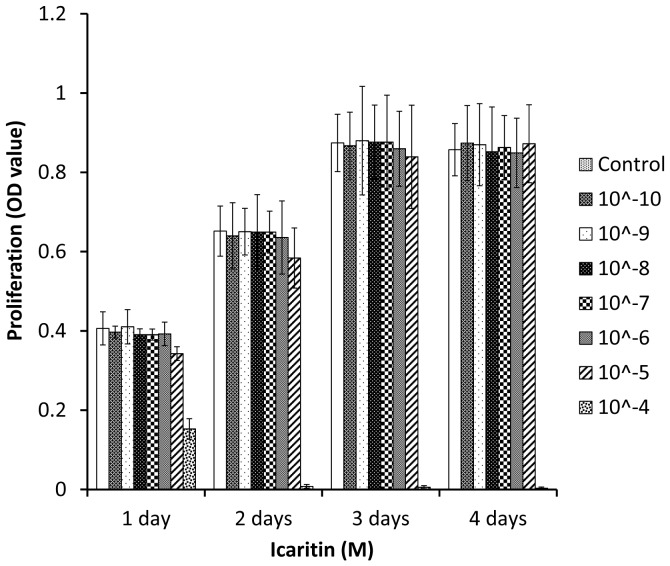
Icaritin did not affect MSCs proliferation at a wide range of doses. The cells were incubated with Icaritin (10∧-10 M to 10∧-4 M) for 1, 2, 3 and 4 days, then MTT assay was performed to test the proliferation ability. DMSO served as control.

Proliferation, matrix maturation and mineralization are sequential processes in the osteogenic differentiation of MSCs [48], [49]. The effect of Icaritin on MSCs proliferation was examined firstly by MTT assay. The results showed that there was no obvious toxicity to MSCs when Icaritin was used at a dose up to 10∧-5 M. However, when the dose reached 10∧-4 M, Icaritin started to show toxic effect (Fig. 2). Based on these observations, Icaritin was used at the dose range of 10∧-8 M to 10∧-5 M for the following evaluations on differentiation of MSCs.

### Icaritin promoted osteogenic differentiation of MSCs only in the presence of osteogenic supplements

**Figure 3:**
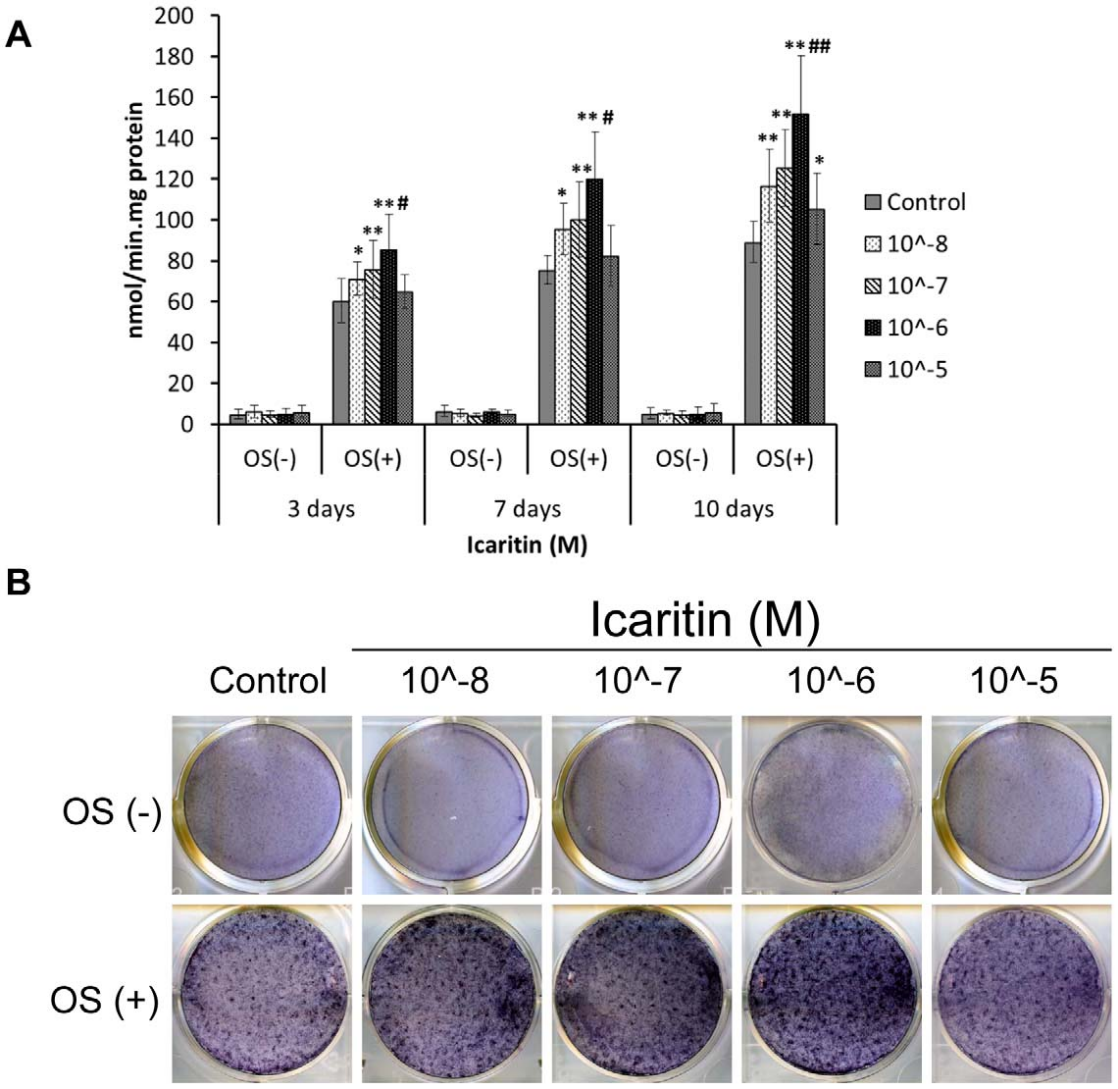
Icaritin increased but not induced alkaline phosphatase (ALP) activity during osteogenic differentiation of MSCs. (A) MSCs treated with Icaritin (10∧-8 M to 10∧-5 M) in absence of OS or in presence of OS for 3, 7 and 10 days respectively, then the cells were lysed and ALP activity assay was performed (OS: osteogenic supplements, ** p<0.01versus control; # p<0.05 and ## p<0.01 versus other group). (B) MSCs were treated the same as in (A) for 10 days, then ALP was stained with BCIP/NBT kit.

ALP is one of the early markers of osteogenic differentiation of MSCs. To study the function of Icaritin in osteogenic differentiation of MSCs, the dose-dependent effects and optimal concentrations need to be determined. In both control group and Icaritin group, ALP activity and expression were detected only in the presence of OS (Fig. 3A, B). The time course study showed temporal changes with presence of Icaritin, with consistent higher ALP level (P<0.01) and on day 10, the ALP activity was strongest and the difference between Icaritin group and DMSO control group was mostly significant (Fig. 3B) (P<0.01). Dose-dependent study revealed that the optimal concentration of Icaritin was at 1 µM (Fig. 3A, B).

### Icaritin enhanced mineralization in osteogenic differentiation of MSCs only in presence of osteogenic supplements

**Figure 4:**
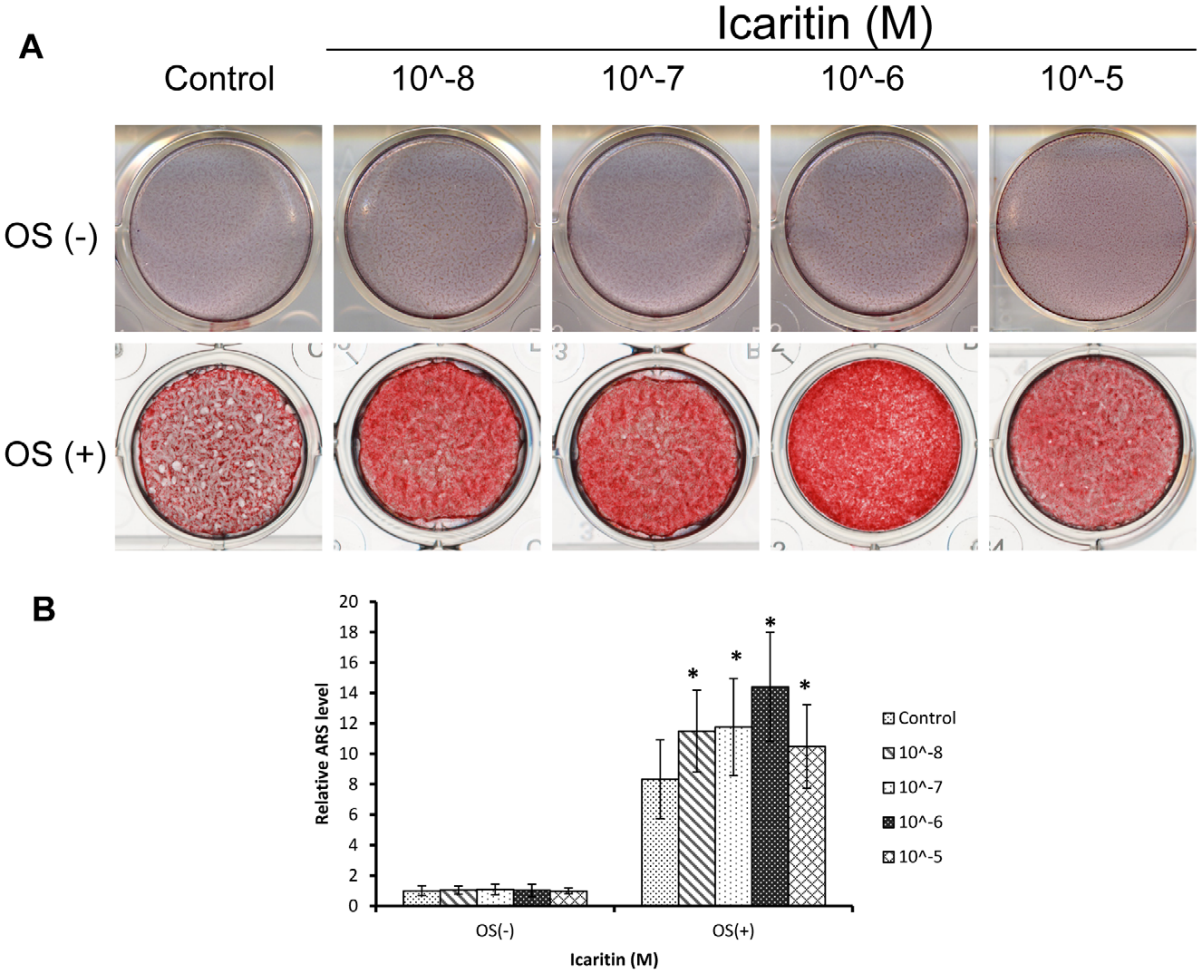
Icaritin promoted but not induced mineralization in osteogenic differentiation of MSC. (A) MSCs treated with Icaritin (10∧-8 M to 10∧-5 M) in absence of OS or in presence of OS for 16 days, then the calcium deposits were stained by Alizarin Red S (ARS). (B) The Alizarin Red S in (A) was eluted by 10% (wt/vol) cetylpyridinium chloride, and the concentrations were determined by absorbance measurement at 562 nm (** p<0.05).

Mineralization is an important functional index of osteogenic differentiation *in vitro* and bone regeneration *in vivo*. Alizarin Red S (ARS) staining showed that at day 16, there was no calcium nodules formation in absence of OS with or without Icaritin treatment; while in presence of OS, there was a large amount of calcium nodules both in the control and Icaritin group (Fig. 4A). Quantification showed that compared with the control,Icaritin significantly increased calcium deposition with the maxima at the concentration of 1 µM (p<0.05, Fig. 4B).

### Icaritin upregulated mRNA expression of osteoblastic marker genes during osteogenic differentiation of MSCs

**Figure 5:**
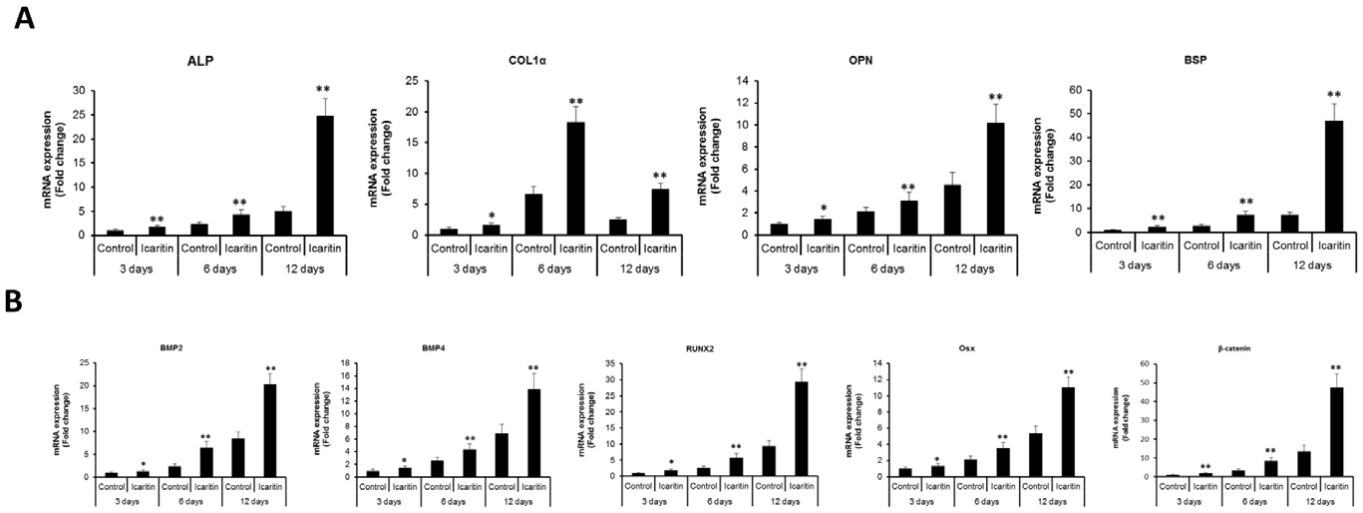
Icaritin upregulated osteoblastic marker genes expression during osteogenic differentiation of MSCs. MSCs cultured in OS medium with or without Icaritin (10∧-6 M) for 3, 6 and 12 days respectively, before the RNA was extracted and Real-time PCR was performed (* p<0.05).

Real-time PCR showed that marker genes including ALP, Col1α, OPN and BSP were up-regulated with Icaritin treatment for 3, 6 and 12 days in the presence of OS, and at day12, ALP, OPN and BSP were increased most, by about 399%, 123% and 519%, respectively, while at day 6, Col1α was the mostly increased one, by about 177% (p<0.01, Fig. 5A). BMPs and Wnt signaling pathways were reported to be involved in osteogenesis of MSCs, so the effect of Icaritin on the BMP signaling pathway was also investigated. In the presence of OS, with Icaritin treatment the mRNA expression level of several BMP related regulators including BMP-2, BMP-4, Runx2 and Osterix (Osx) were increased from day 3 to day 12, and at day 12, they were up-regulated by about 140%, 102%, 214% and 107%, respectively, as well as beta-catenin gene up-regulated by about 252% (p<0.01, Fig. 5B).

### Icaritin inhibited fat droplets formation during adipogenic differentiation of MSCs

**Figure 6:**
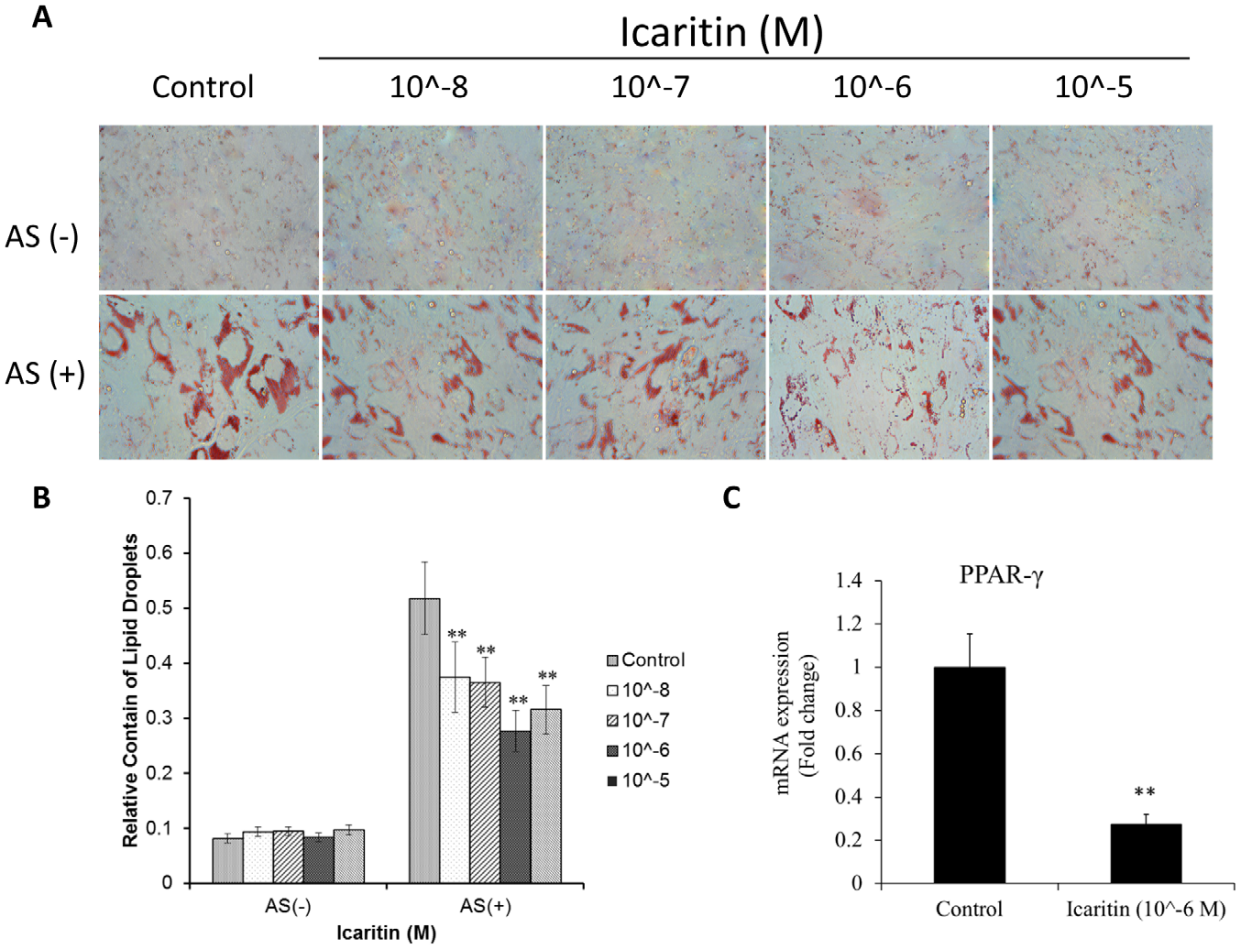
Icaritin inhibited adipogenic differentiation of MSCs. (A) MSCs were cultured in normal or adipogenic differentiation medium with or without Icaritin added for 21 days before Oil Red O staining was performed; (B) Quantification of Oil Red O in (A) at 520 nm OD (** p<0.01); (C) mRNA level of adipogenic gene PPAR-γ (** p<0.01).

The Oil Red O staining showed that Icaritin inhibited the lipid droplets formation in dose-dependent manner in the presence of AS (Fig. 6A). Quantification of Oil Red O staining showed that the lipid droplets were decreased by about 47% when treated with Icaritin at concentration 1 µM (p<0.05, Fig. 6B). Real-time PCR showed that Icaritin with a concentration of 1 µM down-regulated the adipogenic gene PPAR-γ by 265% (p<0.01, Fig. 6C).

### Icaritin had no effect on angiogenesis *in vitro*

**Figure 7:**
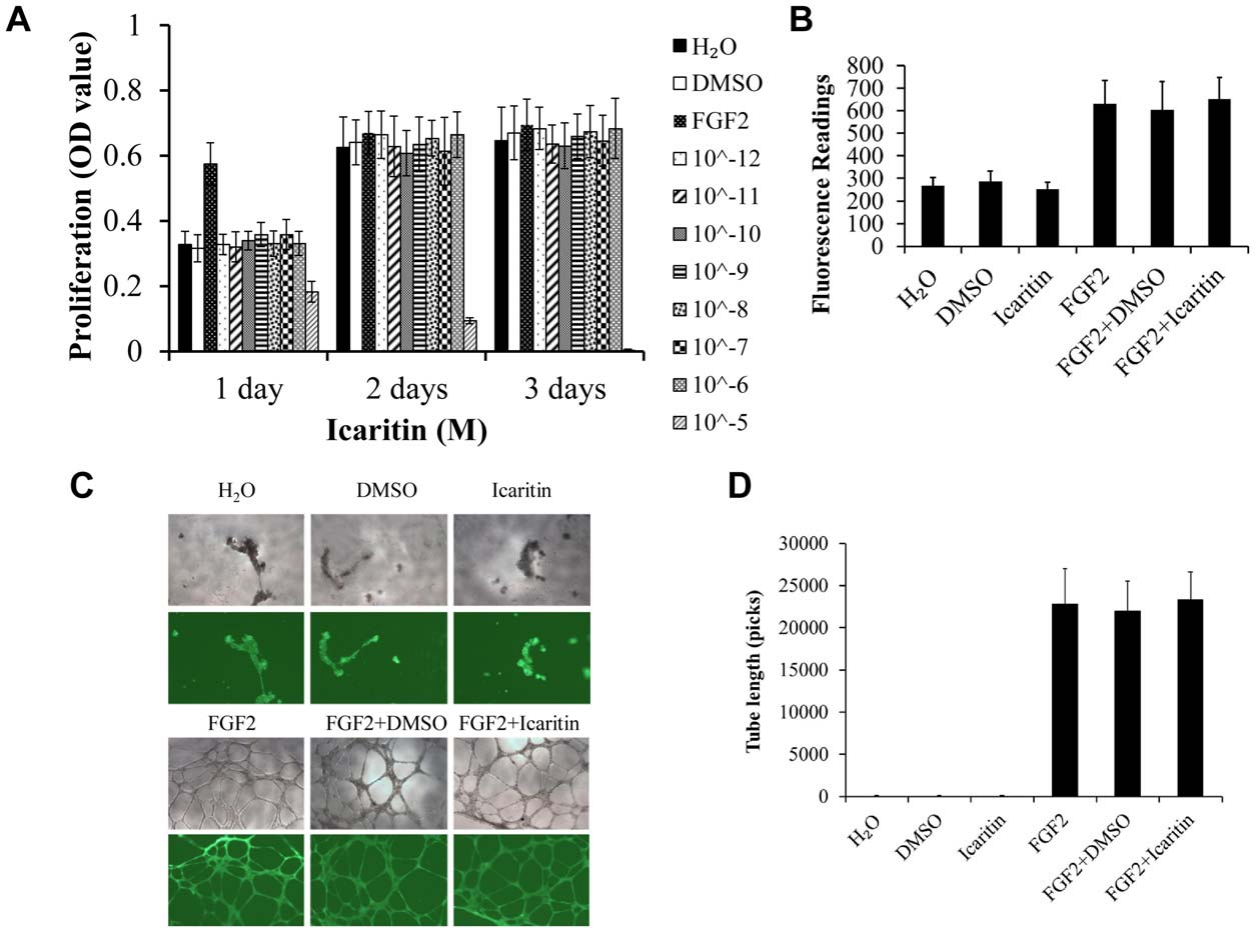
Icaritin did not affect the proliferation, migration, and tube-like structure formation by HUVECs. (A) HUVECs were treated with the indicated concentrations of icaritin for 1, 2 and 3 days, and cell proliferation was determined by MTT assay, H_2_O and DMSO served as negative controls, FGF2 served as positive control. (B) Quantification of chemotatic migration in HUVECs treated with Icaritin (10∧-6 M) or FGF2 in Transwell plates for 12 h. (C) Tube formation in HUVECs cultured on a layer of Matrigel with or without Icaritin (10∧-6 M) or FGF2 for 16 h observed using an inverted phase contract microscope with a video graphic system. (D) Tube length in (C) was quantitated using Image-Pro Plus software.

Angiogenesis is a complex biological process that requires the precise coordination of multiple and various steps, such as endothelial cell proliferation, migration and tube formation. Several groups have reported that very low (e.g., nanomolar or picomolar) concentrations of certain drugs could significantly affect endothelial cell growth [50]–[52]. So in this study, we started HUVECs proliferation assay with lower concentration of Icaritin. At the concentration of 10∧-5 M, Icaritin showed cytotoxicity to HUCECs, while no stimulative or inhibitory effect was found at lower concentrations of a wide range (10∧-12 M to 10∧-6 M) compared with the control (Fig. 7A). Using Transwell plates, it was found that HUVECs migration ability was not changed when treated with Icaritin (10∧-6 M) as compared with either negative or positive control (Fig. 7B). Furthermore, Icaritin (10∧-6 M) didn't affect the tube-like structure formation compared with negative control (DMSO) and positive control (FGF2) (Fig. 7C–D). The quantification for the migration rate and tube length showed that there was no significant difference between Icaritin treated group and the control group, while FGF2 positive control significantly induced migration and tube formation (Fig. 7B–D). In migration and tube formation assay, we also tested a wide range of Icaritin doses from 10∧-12 M to 10∧-6 M, but did not found any promotive or inhibitory effect (Data not shown).

## Discussion

This current study was the first one that systemically demonstrated that a small phytomolecule Icaritin enhanced osteogenic differentiation and inhibited adipogenic differentiation of human bone marrow-derived MSCs *in vitro* that was attributed to its osteopromotive function instead of previously speculated osteoinductive potential. As compared with MSCs derived from other species for studying Icaritin's effects, human-derived MSCs are more relevant for clinical investigations and applications.

In the present study, we started with examination of Icaritin's effect on proliferation of MSCs. We found that Icaritin did not affect the proliferation of MSCs with a wide range of concentrations, except cytotoxicity was tested at the highest concentration in the current study (10∧-4 M). However, if we converted this dose tested *in vitro* into *in vivo*, its serum concentration would be far lower than this *in vitro* dose, implying Icaritin is bio-safety, or non-cytotoxicity to MSCs for *in vivo* applications. In order to determine whether Icaritin promotes osteogenic differentiation of MSCs, early and late osteoblast markers, including ALP and calcium nodule formation – a functional marker of mineralization, were assessed. We found that Icaritin enhanced but not induced osteogenic differentiation of human MSCs. BMP-2 and BMP-4 are known stimulators in osteoblastic differentiation of human MSCs [53]. BMP-2 induces the expression of Runx2, which then regulates the expression of Osx in osteoblastic differentiation [54]–[56]. Real-time PCR analysis showed that RNA levels of BMP2, BMP4, Runx2 and Osx were up-regulated by Icaritin in the presence of OS. These results implied that Icaritin was involved in the BMP signaling pathway in osteogenic differentiation of MSCs. Wnt/beta-catenin plays an important role in MSC osteogenic differentiation, and the up-regulated beta-catenin expression implied that Icaritin enhanced osteogenic differentiation might be associated with Wnt signaling pathway.

ALP activity is used as an early phenotypic marker for mature osteoblasts while the mineralized nodule formation is a phenotypic marker for a later stage of osteogenic differentiation. Our results indicated that Icaritin promoted but not triggered osteogenic differentiation of MSCs from osteoprogenitor stage up to the terminal differentiation stage.

Osteogenesis is negatively coupled with adipogenesis in osteoporosis and osteonecrosis [57]–[60]. We investigated whether Icaritin could affect the adipogenic differentiation of MSCs. The lipid droplets formation under adipogenic induction was also assessed. Oil Red O staining and real-time PCR analysis showed that Icaritin inhibited lipid droplets formation through down-regulation of RNA expression of adipogenic gene PPAR-γ. These results suggested that Icaritin inhibited adipogenic differentiation of MSCs by inhibiting PPAR-γ pathway.

We reported that Icaritin decreased lipid deposition in steroids-associated ON [35], the increased number of small size fat cells in the early steroid-associated ON might be derived from the adipogenic differentiation of MSCs, and this study showed that Icaritin inhibited adipogenic differentiation of MSCs while enhanced osteogenic differentiation of MSCs, on the other hand, Icaritin could re-balance the abnormal differentiation of MSCs. These findings explained the effect of Icaritin on reduction of SAON incidence.

Finally, we examined Icaritin's effect on angiogenesis *in vitro*. Proliferation assay, migration assay and tube-formation assay results showed that Icaritin neither inhibited nor promoted angiogenesis *in vitro*, suggesting no direct inductive effect of Icaritin on angiogenesis.

As bone is a highly vascularized tissue reliant on the close spatial and temporal connection between blood vessels and bone cells to maintain skeletal integrity [60], angiogenesis plays a pivotal role in skeletal development and bone repair. Unlike previous report about Icariin [28], our systemic and controlled *in vitro* study using DMSO as negative control and FGF2 as positive control or its combined treatment did not provide evidences to support that Icaritin was able to trigger or impair angiogenesis. Besides FGF2, vascular endothelial growth factor (VEGF) is also well known as an important molecule in angiogenesis as it can induce angiogenesis via a direct effect on endothelial cells [61], [62]. In this study, we also tested if Icaritin would have agio-promotive effect on VEGF-induced angiogenesis and our unpublished data showed the same results as that of FGF2.

Our previous study showed that Icaritin could reduce incidence of SAON in rabbit model [35]. Recently, we developed osteoconductive porous poly(l-lactide-co-glycolide)/tricalcium phosphate (PLGA/TCP) to incorporate Icaritin to form a PLGA/TCP/icaritin composite scaffold material. This composite scaffold showed good biocompatibility, osteoconduction, interconnectivity, and mechanical properties that were comparable to that of trabecular bone while the bioactivity of Icaritin was still maintained [63].

In conclusion, the results of the present experimental study demonstrated that Icaritin was a safe phytomolecule that possessed osteopromotive but not osteoinductive potentials. Icaritin promoted osteogenic but inhibited adipogenic differentiation of MSCs by regulating osteogenesis and adipogenesis related gene expressions. Icaritin did not trigger angiogenesis or impair angiogenesis *in vitro*. Considering the above-mentioned characteristics of Icaritin and its cost-effectiveness, Icaritin might be considered as a good choice as an osteopromotive phytomolecule for bone tissue engineering.
